# Preparation of lignosulfonate‐based nanofiltration membranes with improved water desalination performance

**DOI:** 10.1002/elsc.202000102

**Published:** 2021-04-02

**Authors:** Wangqu Liu, Xin Geng, Saisai Li, Xia Zhan, Jiding Li, Luying Wang, Jiandu Lei

**Affiliations:** ^1^ Beijing Key Laboratory of Lignocellulosic Chemistry Beijing Forestry University Beijing P. R. China; ^2^ Department of Chemical and Biomolecular Engineering Johns Hopkins University Baltimore MD USA; ^3^ Key Laboratory of Cleaner Production and Integrated Resource Utilization of China National Light Industry Beijing Technology and Business University Beijing P. R. China; ^4^ State Key Laboratory of Chemical Engineering Department of Chemical Engineering Tsinghua University Beijing P. R. China

**Keywords:** layer‐by‐layer self‐assembly, nanofiltration, polyethylenimine, sodium lignosulfonate, water desalination

## Abstract

Pulping and papermaking generate large amounts of waste in the form of lignosulfonates which have limited valorized applications so far. Herein, we report a novel lignosulfonate‐based nanofiltration membrane, prepared by using polyethylenimine (PEI) and sodium lignosulfonate (SL) via a layer‐by‐layer (LbL) self‐assembly. As a low‐cost and renewable natural polyelectrolyte, SL is selected to replace the synthetic polyelectrolyte commonly used in the conventional LbL fabrication for composite membranes. The prepared LbL (PEI/SL)_7_ membranes were crosslinked by glutaraldehyde (GA) to obtain (PEI/SL)_7_‐GA membranes with compact selective layer. We characterized (PEI/SL)_7_ and (PEI/SL)_7_‐GA membranes to study the chemical compositions, morphologies, and surface hydrophilicity. To improve the nanofiltration performances of the (PEI/SL)_7_‐GA membranes for water desalination, we investigated the effects of the crosslinking time, GA concentration and the NaCl supporting electrolyte on membrane structure and performance. The optimized (PEI/SL)_7_‐GA membrane exhibited a permeating flux up to 39.6 L/(m^2^·h) and a rejection of 91.7% for the MgSO_4_ aqueous solution 2.0 g/L concentration, showing its promising potential for water desalination. This study provides a new approach to applying the underdeveloped lignin‐based biomass as green membrane materials for water treatment.

**Abbreviations**
GA: glutaraldehyde
LbL: layer‐by‐layer
NF: nanofiltration
PEI: polyethylenimine
SL: sodium lignosulfonate
TFC: thin‐film composite

## 1 INTRODUCTION

Nanofiltration (NF) has been considered a green membrane separation technology is owing to its high flux, mild operation conditions, and energy‐saving [1, 2]. Taking these advantages, NF plays a critical role in water treatment and industrial fields because it selectively rejects multivalent ions and low molecular weight organic compounds [3, 4, 5]. Most commercial NF membrassnes are thin‐film composite (TFC) membranes fabricated with an ultrathin selective layer coating on a porous substrate, which can be fabricated through the coating, interfacial polymerization, photo‐grafting, and layer‐by‐layer (LbL) methods [6, 7, 8, 9]. Among these TFC NF membranes fabrication methods, LbL is widely used owing to its tailorable thicknesses and surface charges of selective layers by varying polyelectrolytes, assembly layers, or other fabrication conditions [10].

To date, the polyelectrolytes reported for preparing TFC NF membranes are mostly industrial synthetic polyelectrolytes, such as polyacrylic acid, polyethylenimine (PEI), polyvinylamine, poly(allylamine hydrochloride), poly(diallyldim‐ethyl ammonium chloride), polystyrene sulfonate, and polyvinyl sulfate [11, 12, 13, 14, 15]. These synthetic polyelectrolytes originate from non‐renewable raw materials and are produced by industrial chemical processing, which consumes a considerable amount of energy and may generate harmful waste. To reduce the use of synthetic polyelectrolytes in LbL NF membranes fabrication, some recent works focused on making LbL NF membranes from nature‐originated polyelectrolytes, such as sodium alginate, carboxymethyl cellulose, and chitosan [16, 17, 18, 19, 20]. However, these materials need purification and modification processing from their raw materials, which usually increases the manufacturing costs.

**PRACTICAL APPLICATION**

This study reported a new approach for fabricating sustainable lignosulfonate‐based nanofiltration membranes utilizing pulping waste for water desalination. The novel thin‐film composite nanofiltration membranes were prepared through layer‐by‐layer self‐assembly by using sodium lignosulfonate (SL) and polyethylenimine (PEI) as polyelectrolytes. The influences of fabrication conditions on membrane structure and nanofiltration performance were investigated for improving water desalination performance. It shows that the lignosulfonate‐based membrane has a promising potential for water desalination and the method also opens a new approach for lignosulfonate biomass valorization.

Lignosulfonates are a group of natural polyelectrolytes produced as by‐products of the sulfite pulping process in the papermaking industry. Unlike the previously mentioned natural polymers and their derivatives, which require extra processing steps. Lignosulfonates are low‐cost by‐products can be obtained directly from pulping factories with millions of tons of production per year [21]. They consist of a long hydrophobic chain and branched hydrophilic and ionizable side groups such as carboxyl and sulfonate groups, the basic molecular formulas of lignin and lignosulfonates are shown in Figure S1. Due to the negatively charged sulfonate groups, lignosulfonates are considered natural polyanions [22]. Pulping conditions determine the different types of ionizable groups in the lignosulfonates, which mostly consist of sodium, calcium, and magnesium [23, 24, 25]. Most lignosulfonates are just burned for energy generation in the pulping processes [23]. The valorized applications of lignosulfonates are limited now [26, 27]. Several recent works have reported the application of sodium lignosulfonate (SL) as a natural polyelectrolyte through LbL self‐assembly. These researches mostly built a multilayered structure on a supporting matrix by alternatively depositing polyelectrolytes with opposite charges, in order to improve the hydrophobic, mechanical, and thermal properties of the coated materials, such as fibers, papers, and foams [28, 29, 30, 31, 32].

In this study, we reported SL‐based TFC NF membranes prepared by using LbL deposition of PEI/SL selective layers and followed by a chemical crosslinking using glutaraldehyde (GA) to enhance the performance of selective layers. To improve the NF performance of the (PEI/SL)_7_‐GA membranes, we evaluated the influence of crosslinking conditions and/or supporting electrolyte on the membrane structures, morphologies and NF performances for water desalination. This work merges the advantages of lignin‐based biomass into NFM separation technology and provides a new approach to valorize the underdeveloped lignosulfonates resources into scalable, low‐cost fabrication methods of NF membranes for water treatment.

## 2 MATERIALS AND METHODS

### 2.1 Materials

Polysulfone (PSF) ultrafiltration membranes substrates (MWCO: 30 kDa) were obtained from Beijing Pureach Tech. Ltd. (China). Sodium lignosulfonate (SL, 96% purity) and polyethylenimine (PEI, Mw = 70000 Da, 50% in water) were purchased from Yuanye Biotech Co., Ltd. (Shanghai, China). Glutaraldehyde (GA, 50% in water), NaCl, and MgCl_2_ were purchased from Sinopharm Chemical Reagent Co., Ltd. (Shanghai, China). NaOH and MgSO_4_ were purchased from Xilong Scientific Co., Ltd. and Tianjin Jinke Fine Chemical Research Institute, respectively. All the chemicals and solvents were of or above reagent grade, and they are used as received without further purification. DI water was used for all experiments.

### 2.2 Membrane fabrication

**FIGURE 1:**
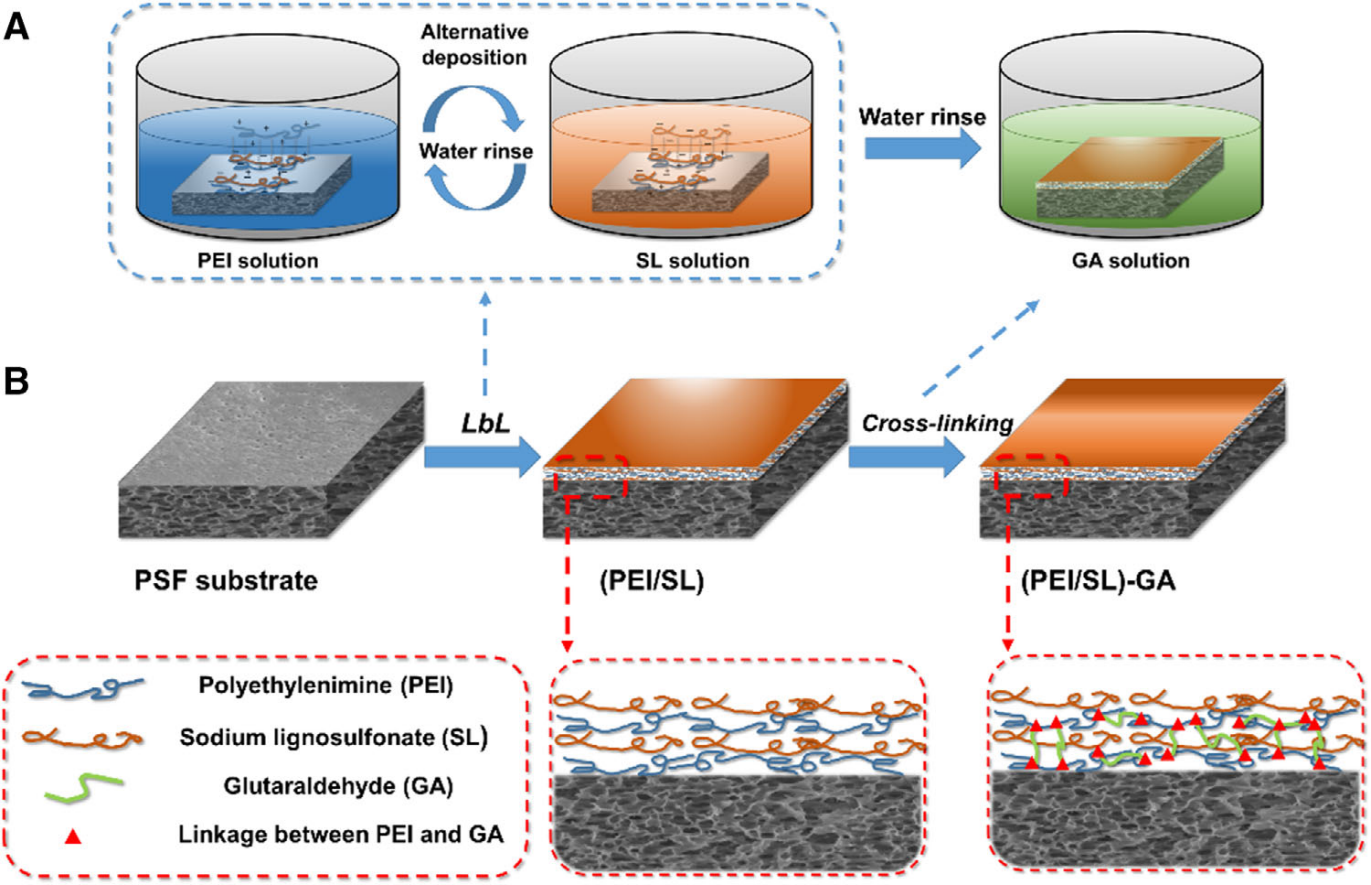
(A) Schematics of the SL‐based NFM fabrication, including the LbL deposition of polyelectrolyte and chemical crosslinking. (B) Schematics showing the structure of PSF substrate, (PEI/SL)_n_ multilayer‐coated membrane and crosslinked (PEI/SL)_n_‐GA NFM

The PSF membrane was firstly immersed into a PEI polycation aqueous solution (0.20 wt%) and rinsed by DI water, then the PEI/PSF membrane was immersed into an SL polyanion aqueous solution (0.30 wt%) and rinsed by DI water, giving a pair of (PEI/SL) layer. This procedure was repeated at room temperature until reaching seven pairs of the polyelectrolyte bilayers. The prepared (PEI/SL)_7_ membrane was immersed into a GA solution to take place a typical crosslinking reaction, in which the Schiff's base reaction took placed between amino groups of PEI and aldehyde groups of GA molecules on the TFC membranes (as shown in Figure S2). The LbL self‐assembly procedures is shown in Figure 1. A serial of (PEI/SL)_7_‐GA membranes were prepared by varying the number of bilayers, the crosslinking time, and the concentration of the NaCl supporting electrolyte in the PEI or SL solutions.

### 2.3 Characterization experiments

The surface chemical properties of the substrates and the composite membranes were examined by Nicolet 6700 ATR‐FTIR spectroscopy (Thermo Fisher Scientific, USA). The spectra were collected by 32 scans in the range of 400 to 4000 cm^−1^ at a resolution of 4 cm^−1^. The surface Zeta potential of composite membranes was measured by a SurPASS electrokinetic analyzer (Anton Paar, Austria) equipped with a clamping cell at 300 mbar using 1 mM KCl solution as the electrolyte solution. The morphologies of the surface and cross‐sections of the substrates and composite membranes were observed by an S8010 scanning electric microscopy (SEM; Hitachi, Japan). The cross‐sectional samples were prepared by fracturing the membranes in liquid nitrogen. Before observation, all samples were gold‐coated under vacuum conditions. The 3D surface morphologies and roughness of the membranes were obtained by a MultiMode 8 atomic force microscopy (AFM; BRUKER, USA) with the measuring size of 4×4 μm along the X, Y‐axis using ScanAsyst mode. The surface water contact angles of substrates and composite membranes were measured with an SL200KS optical contact angle goniometer (Kino industry Co., Ltd., China) by sessile drop method.

### 2.4 NF performance tests

All NF experiments were conducted by a WTM‐0806H UF/NF performance test system produced by Hangzhou Watech Co., Ltd., with an effective membrane area of 69.40 cm^2^. The schematic of this system is shown in Figure S3. A flat membrane is placed in the crossflow membrane cell pressurized by a pump. The NF experiments used 2.0 g/L salt solutions or 0.1 g/L dye solutions as feeds to examine the separation performance of the (PEI/SL)‐GA membranes. The feed flow was constantly circulated from the feed tank to the membrane cell with a steady operating pressure of 1.0 MPa in each test. Before collecting permeate samples, the system was pre‐operated at least 1 h until reaching a steady condition. For each kind of membrane, at least three membranes were evaluated. The permeate flux (*J*) was calculated by Equation (1); the rejection (*R*) was calculated by Equation (2):

(1) $$J\;=\frac{V}{A\cdot t}\;$$

(2) $$R\;=\frac{C_{f}-C_{p}}{C_{f}}\;\times 100\%$$

where *V*, *A*, and *t* represent the volume of permeate (L), the effective membrane area (m^2^), and the permeation time (min) respectively; the *C_f_* represents the solute concentration in the feed (g/L), and the *C_p_* represents the solute concentration in permeate (g/L). The solute concentrations were measured by an electrical conductivity meter (DDS‐307A, INESA Instrument, China) and an ultraviolet‐visible spectrophotometer (N5000, shanghai Yoke Instrument, China) for salts and dyes, respectively.

## 3 RESULTS AND DISCUSSION

### 3.1 Characterization results of (PEI/SL)_7_ and (PEI/SL)_7_‐GA membranes

**FIGURE 2:**
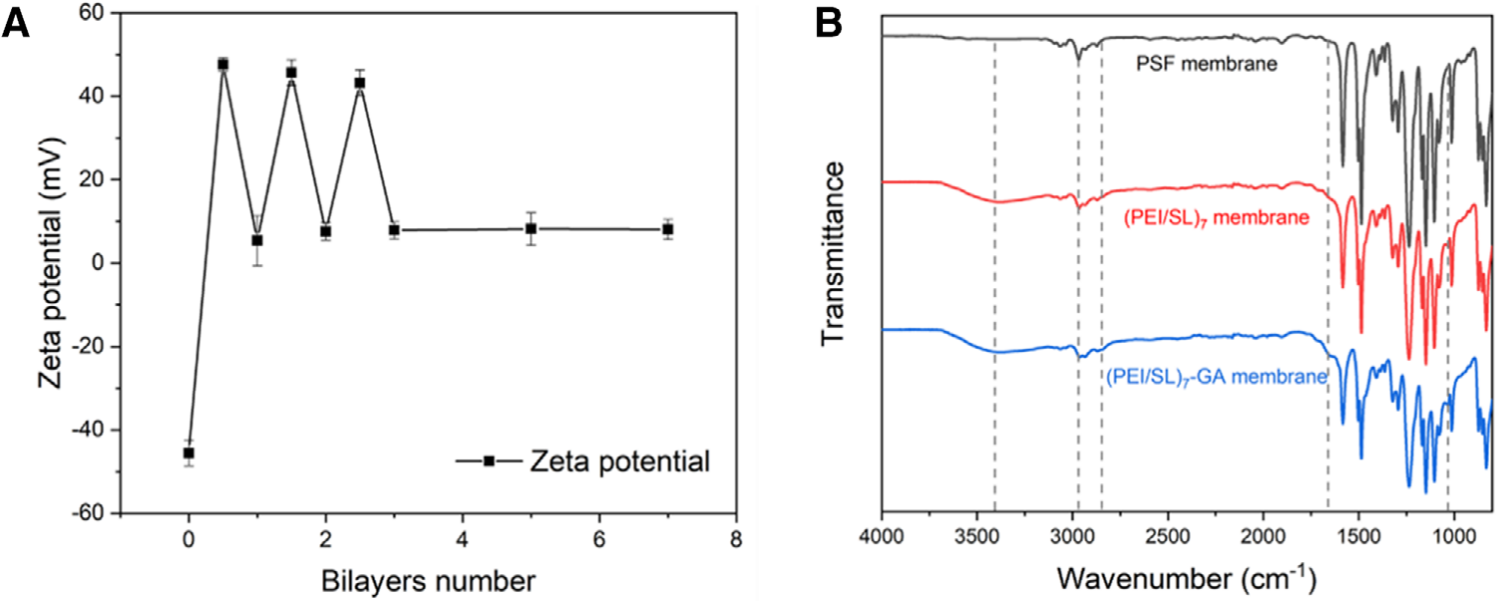
(A) Plot of zeta potential values of (PEI/SL)_n_ LbL membranes with respect to the number of deposited polyelectrolyte layers. The layer numbers ending with 0.5 represent a membrane with a PEI top layer. (B) FTIR spectra of PSF, (PEI/SL)_7_ (PEI/SL)_7_‐GA membranes

To study the polyelectrolyte LbL self‐assembly process, we monitored the surface zeta‐potential values of the (PEI/SL)_7_ membranes during the alternative polyelectrolyte deposition. As shown in Figure 2A, the zeta‐potential value alternatively increased and decreased, which indicates the self‐assembly of PEI and SL took place in an LbL manner, and the prepared (PEI/SL)_7_ membrane was positively charged. We observed that the negatively charged PSF substrate had a zeta‐potential of ‐45.6 mV. After deposition of the first layer of PEI, the PEI membrane became positively charged with the zeta‐potential of 47.5 mV. Then the zeta‐potential of (PEI/SL)_1_ membrane with the first layer of SL decreased to 5.38 mV, but the value did not drop to negative due to the relatively weak electronegativity of SL. The zeta‐potential values of membrane surfaces fluctuated up and down with a decreasing absolute value. When the number of (PEI/SL) bilayers increased beyond 5 up to 7, the zeta‐potential values of the (PEI/SL) membrane all stabilized around 8 mV.

The total reflectance (ATR)‐FTIR spectroscopy of virgin PSF, (PEI/SL)_7_ membranes are shown in Figure 2B. The IR spectra of the (PEI/SL)_7_ membrane (red line) shows the following features of PEI and SL compared with the PSF substrate, which confirmed the successful deposition of (PEI/SL) multilayers on the substrate: it has a stronger peak at *v* = 3200–3600 cm^–1^ than the PSF membrane which corresponds to the N‐H and ‐OH stretching vibration of the newly deposited PEI and SL. Also, the slightly strengthened peaks at *v* = 2930 cm^–1^ and *v* = 1650 cm^–1^ both indicate the vibrations of C‐H stretches on –CH_2_– groups in both PEI and SL [33, 34]. A new peak at *v* = 1030 cm^–1^ represents the S=O symmetric stretching of sulfonate in SL. Compared with the spectroscopy of virgin PSF substrate, these new arising and strengthened peaks indicate the successful deposition of (PEI/SL) multilayers on the substrates. The blue line in Figure 2A shows the IR spectra of (PEI/SL)_7_‐GA membrane, which has similar features to the spectra of (PEI/SL)_7_ membrane (red line), indicating the GA crosslinking does not significantly vary the polyelectrolytes multilayer structure. Several peak changes are observed as the results of the crosslinking reaction. For example, the strengthening of the peaks at *v* = 2840 cm^–1^ and 2930 cm^–1^ corresponds to the symmetrical stretching vibration of more –CH_2_– structures induced by GA molecules [35, 36]. A significant peak strengthening at *v* = 1650 cm^–1^ corresponds to the stretching vibration of an imine (C=N) stretching as a result of the formation of the Schiff's base structures during the PEI‐GA crosslinking reaction.

**FIGURE 3:**
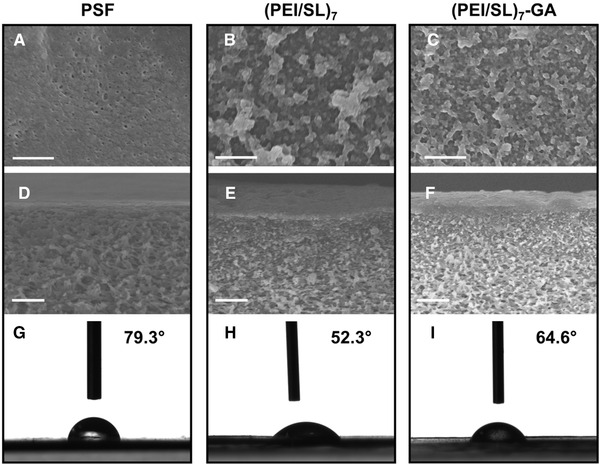
Surface SEM images of (A) PSF, (B) (PEI/SL)_7_ and (C) (PEI/SL)_7_‐GA membranes (each scale bar represents 0.5 μm). Cross‐sectional SEM images of (D) PSF, (E) (PEI/SL)_7_ and (F) (PEI/SL)_7_‐GA membranes (each scale bar represents 1.0 μm). Surface water contact angle images of (G) PSF, (H) (PEI/SL)_7_ and (I) (PEI/SL)_7_‐GA membranes

The surface and cross‐sectional SEM images of the virgin PSF membrane (Figure 3A,D) show uniformly distributed submicron‐sized pores on the surface and spongy like structures at the cross‐section, a morphology consistent with PSF ultrafiltration membranes substrate. The surface (PEI/SL)_7_ membrane (Figure 3B) shows an uneven surface with bumps. The polyelectrolytes multilayer covered on the PSF substrate makes the pores on the PSF substrate invisible. We infer that the (PEI/SL)_7_ membrane surface bumps are coiled SL polymer chain and SL nanoparticles. Since SL is a weak polyanion which trends to coil up in water dispersion, and the strong amphiphilicity of SL molecule facilitates the coil‐to‐globule transition from coiled up chain to nanoparticles [37, 38]. After crosslinking, the surface of (PEI/SL)_7_‐GA (Figure 3C) shows a more compact morphology with bumps in reduced size. The cross‐sectional image of the (PEI/SL)_7_ membrane (Figures 3E) shows a TFC structure with an ultrathin layer of a nanoscale thickness coated on the porous and spongy PSF substrate. The (PEI/SL)_7_‐GA membrane (Figure 3F) has a similar TFC structure after crosslinked by GA. While the thickness of the selective layer of the crosslinked (PEI/SL)_7_‐GA membrane tends to be denser and slightly thinner than the uncross‐linked (PEI/SL)_7_ membrane. A possible explanation for these morphologies changes is that the crosslinking between the PEI layer compressed the selective layer, therefore creates a more compact layer with decreasing thickness [37].

The surface wettability of (PEI/SL)_7_ (Figure 3H) improved compared to the virgin PSF substrate (Figure 3G) due to the introduction of the hydrophilic groups in the polyelectrolytes layer. The water contact angle decreased from 79.3° to 52.3°. The surface wettability of (PEI/SL)_7_‐GA (Figure 3I) decreased to 64.6°, since the crosslinking reaction consumed part of the hydrophilic amino groups and induced new hydrophobic methylene groups. The change of surface roughness might also contribute to the surface wettability changes.

**FIGURE 4:**
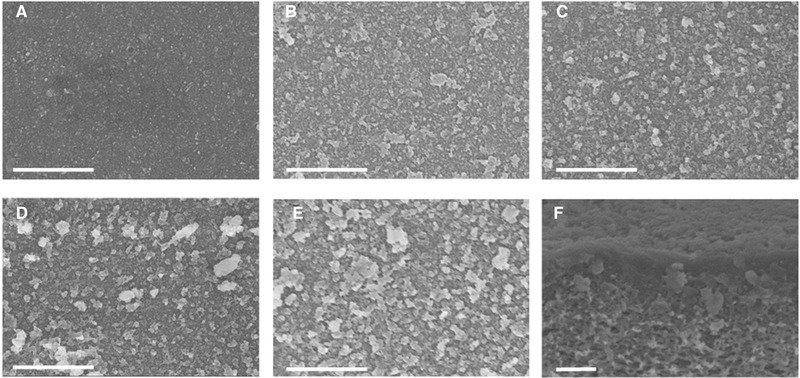
(A‐E) Surface SEM images of the (PEI/SL)_7_‐GA membranes prepared at the NaCl concentration of 0, 0.25, 0.50, 0.75, and 1.0 M, respectively (each scale bar represents 2.0 μm); (F) cross‐sectional SEM image of the (PEI/SL)_7_‐GA membrane prepared at the NaCl concentration of 0.25 M, the scale bar represents 1.0 μm

The (PEI/SL)_7_‐GA membranes prepared adding supporting electrolytes (NaCl) into PEI and SL solutions were also be characterized by SEM and water contact angle measurements. Figure 4 shows the effect of supporting electrolyte NaCl concentration on membrane surface morphology. The surface of the (PEI/SL)_7_‐GA membrane without adding NaCl supporting electrolytes is relatively smooth and uniform. When the supporting electrolyte is added, the surface of the (PEI/SL)_7_‐GA membrane begins to become rougher with larger bumps (Figure 4D‐E). The cross‐sectional SEM images of the (PEI/SL)_7_‐GA membranes with NaCl supporting electrolyte (Figure 4F) exhibit the morphology and thickness similar to the one without supporting electrolyte (Figure 3F). To conclude, adding supporting electrolytes in the LbL fabrication can vary the morphology of the membrane surface but cannot significantly change the membrane thickness. Moreover, the water contact angles of (PEI/SL)_7_‐GA membranes affected by NaCl concentrations are listed in Table S1. It can be found that the membrane surface contact angles tend to decrease from 64.6° to 55.5°–57.6°, mainly caused by the rougher surface after adding NaCl supporting electrolytes. Overall, the membrane morphology and hydrophilicity cannot change significantly with an increasing NaCl concentration.

The membrane morphology change during the polyelectrolytes self‐assembly process that results from adding supporting electrolytes in the polyelectrolyte solution can be explained by the charges on polymer chains that can be balanced “intrinsically” by oppositely charged chains or be balanced “extrinsically” by salt counter‐ions within the polyelectrolyte solution [39]. In this case, the NaCl in the PEI and SL solutions improved the “extrinsic” compensation and weakened “intrinsic” compensation. Therefore, the PEI and SL polymer chains are coiled up with the presence of NaCl, resulting in the morphology and structure change of the polyelectrolyte multilayers. Generally, adding supporting electrolyte contributes to a rougher and looser polyelectrolyte multilayer because of the less interpenetrated polyelectrolyte chains [40].

### 3.2 Effect of crosslinking conditions on nanofiltration performance of (PEI/SL)_7_‐GA membranes

The crosslinking reaction between the (PEI/SL)_7_ multilayer and GA creates a compact selective layer. We evaluated the NF performance of (PEI/SL)_7_‐GA membranes fabricated under different crosslinking conditions. All the NF performance tests were carried out under an operating pressure of 1.0 MPa with a 2.0 g/L MgSO_4_ solution feed.

**FIGURE 5:**
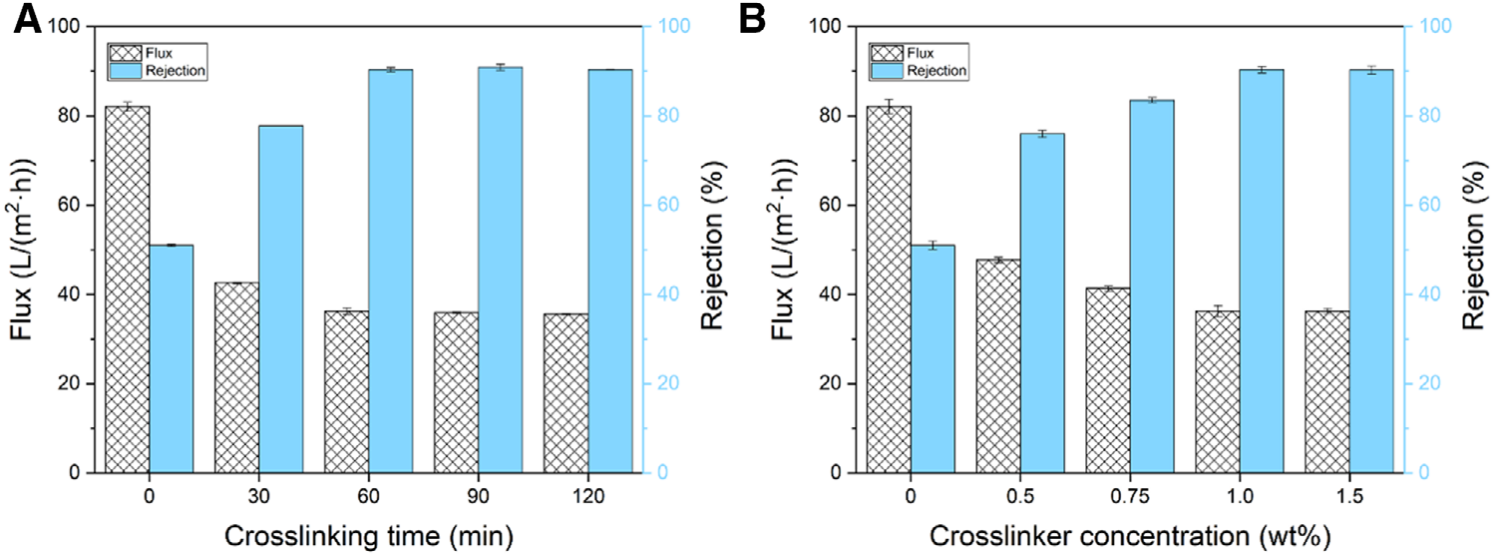
The effect of crosslinking conditions on NF performance of (PEI/SL)_7_‐GA membranes: (A) time of crosslinking treatment, (B) crosslinker (GA) concentrations

The crosslinking time determines the progress of the crosslinking reaction, which results in different degrees of crosslinking for the (PEI/SL)_7_‐GA selective layers, and influence the NF performance of the NFMs. To study the effect of crosslinking time on NF performance of (PEI/SL)_7_‐GA membranes, we fabricated a series of (PEI/SL)_7_‐GA membranes using the same PEI and SL solutions as mentioned above, while crosslinked by 1.0 wt% GA for different times (0–120 min). The results of NF performance changing with crosslinking time are shown in Figure 5A. Overall, the membranes exhibit improving rejection and reducing flux, with the elongation of crosslinking time. This trend can be explained by the fact that the longer crosslinking time results in a more sufficient crosslinking between PEI and GA, which creates a denser selective layer with improved rejection and reduced water permeability. The uncross‐linked (PEI/SL)_7_ membrane shows the lowest rejection of 51.4% but the highest 82.1 L/(m^2^·h) flux due to its relatively loose selective layer. After 30 min crosslinking, the water flux dropped to 42.6 L/(m^2^·h), while the rejection raised to 77.8%. This rejection just reached the standard for effective inorganic salt separation, which should have a rejection of around 90%. The (PEI/SL)_7_‐GA membrane crosslinked less than 30 min might have potential for organic solute separation. When the crosslinking time reaches 60 min, the NF performance of (PEI/SL)_7_‐GA reaches a critical point with a rejection of 90.3% and flux of 36.3 L/(m^2^·h). After this point, the rejection does not increase remarkably, while the flux keeps going down slightly. It means that the crosslinking reaction between the GA and PEI macromolecules reached an equilibrium state at about 60 min, so the increase in crosslinking time beyond the critical point will not promote the degree of crosslinking.

The crosslinker concentration also influences the process of the crosslinking reaction, which subsequentially changes the (PEI/SL)_7_‐GA selective layer structure and determines the NF performance of the membranes. To study the effect of crosslinker concentration on NF performance of (PEI/SL)_7_‐GA membranes, the GA concentration was varied from 0 wt% to 1.5 wt% for 60 min. The results of NF performance varying with different crosslinker concentrations are shown in Figure 5B. Overall, the rejection increases with the increase of GA concentration, while the permeant flux decrease. The control (PEI/SL)_7_ membrane treated with DI water without any GA shows the lowest rejection of 51.4% but the highest 82.1 L/(m^2^·h) flux due to uncross‐linked loose selective layer. The increase of rejection and decrease of flux reached a plateau at 1.0 wt% GA concentration. The (PEI/SL)_7_‐GA treated by 1.0 wt% GA exhibited a rejection of 90.3% and flux of 36.3 L/(m^2^·h). After this point, the rejection remains at around 90.3%, while the flux keeps going down slightly. Generally, the higher concentration of cross‐linker results in more crosslinking reactions and then forms a denser selective layer, which rejects more solutes and reduces water molecule permeability. The crosslinking reaction between the GA and PEI reached an equilibrium when the crosslinker concentration go up to 1.0 wt%, where the crosslinking reaction will not significantly move forward with the increasing GA concentration. Thus, the optimal crosslinking conditions are the crosslinking time of 1 h and the GA concentration of 1.0 wt%.

### 3.3 Effect of supporting electrolyte on nanofiltration performance of (PEI/SL)_7_‐GA membranes

To further improve the NF performance of the (PEI/SL)_7_‐GA membranes, we added NaCl as a supporting electrolyte to the polyelectrolyte solutions. It has been reported that adding inorganic salts in the polyelectrolytes solutions as a supporting electrolyte can significantly change the polyelectrolyte multilayer structure and separation performance by influencing the intermolecular electrostatic interactions [10, 40]. A series of the (PEI/SL)_7_‐GA were prepared by adding NaCl solution with concentrations ranging from 0 to 1.0 M as the supporting electrolyte into both PEI and SL solutions. Other fabrication conditions were the same as previous tests, using 0.20 wt% of PEI, 0.30 wt% of SL, and 1.0 wt% GA crosslinked for 60 min.

**FIGURE 6:**
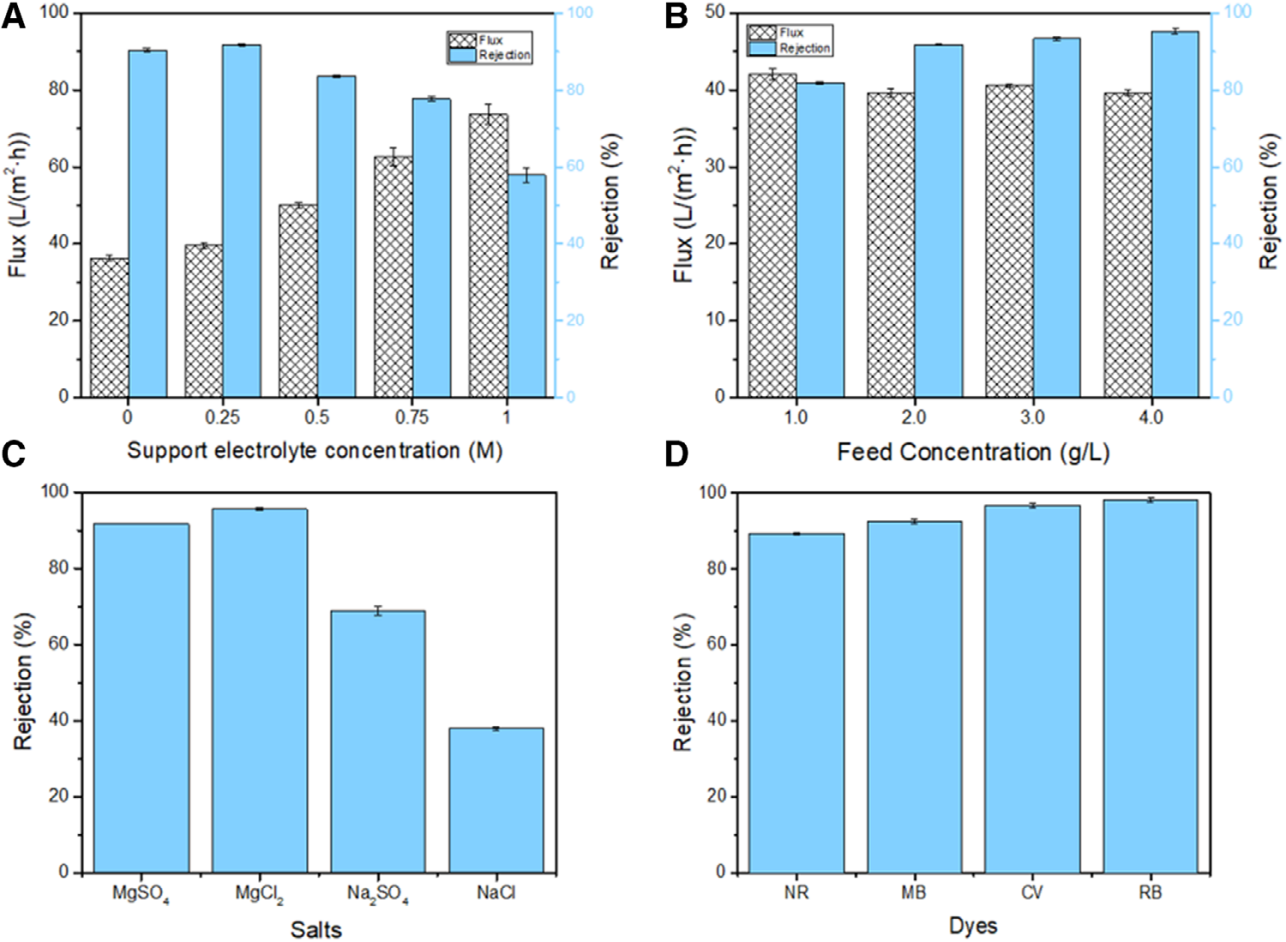
(A) Effect of NaCl support electrolyte concentrations on NF performance of (PEI/SL)_7_‐GA membranes, (B) Effect of feed concentration on MgSO_4_ rejection of the optimized membrane, (C, D) rejections of the optimized membrane for different salts and organic dyes

The NF performance of (PEI/SL)7‐GA membranes prepared with different concentrations of NaCl as supporting electrolyte is shown in Figure 6. For the (PEI/SL)_7_‐GA membrane modified by adding 0.25 M NaCl as supporting electrolytes, the flux increases from 36.3 to 39.6 L/(m^2^·h), and the rejection slightly increases from 90.3 to 91.7%. When we keep increasing the NaCl concentration from 0.25 to 1.0 M, the flux increases to 73.6 L/(m^2^·h), but the rejection gradually drops to lower than 60%. This result is consistent with previously reported literature, which modified the polyelectrolyte LbL membrane performance by adding supporting electrolyte [40]. Generally, the polyelectrolyte chains coiled together with the presence of supporting electrolyte, which results in a thicker but looser polyelectrolyte coating with higher flux and lower rejection. We observed consistent characterization results in Figure 4 and Table S1, they indicate that the (PEI/SL)_7_‐GA membranes show increasing roughness and wettability with rising NaCl concentration. The rougher and bumpier membrane surface corresponds to the thicker and looser membrane structures caused by increasing salinity. The increase in both flux and rejection at 0.25 M NaCl can be explained by when the concentration of NaCl supporting electrolyte concentration is relatively low. The polyelectrolyte chains coiled slightly; as a result, the slightly loose (PEI/SL)_7_‐GA layer makes water transport easier without comprising on rejection. In contrast, once the NaCl concentration is too high, the polyelectrolyte chains significantly coiled up, resulting in a highly loose membrane with higher water and salt permeability. A good NFM should have relatively high flux and rejection at least higher than 90%; therefore, the optimized supporting electrolyte NaCl concentration for (PEI/SL)_7_‐GA membrane is 0.25 M.

The NF performance of optimized (PEI/SL)_7_‐GA membranes for MgSO_4_ with different feed concentrations were further evaluated. The results are shown in Figure 6B, the (PEI/SL)_7_‐GA membrane showed a gradually increased rejection rate for MgSO_4_ with the increasing feed concentration from 1.0 to 4.0 g/L, the fluxes remain constant at around 40 L/(m^2^·h). The highest rejection reached 95.3% when separating the 4.0 g/L MgSO_4_ solution. This high rejection in high feed concentration is rarely observed in NF membranes, the salt rejection normally decreases with the raising of feed solution concentration. We suggest a reason for this phenomenon is because many salt particles accumulate on the surface under high concentration conditions, adsorbed in the pores of the membrane lead to the pores’ size descended then increased the rejection. This result indicates our (PEI/SL)_7_‐GA membrane has the prospective application in high concentration salt solutions separation.

### 3.4 Performance of the optimized (PEI/SL)_7_‐GA membrane

The NF performance of the optimized (PEI/SL)_7_‐GA NF membrane was measured by using different feeds separately, to investigate the rejection of four inorganic salts and four organic dyes. Figure 6C shows the salt rejections for MgSO_4_, MgCl_2_, Na_2_SO_4_, and NaCl solutions under the same operating conditions (the feed concentration of 2.0 g/L and the operating pressure of 1.0 MPa). The salt rejection values are in the order of 95.6% > 91.7% > 68.8% > 37.9% from MgSO_4_, MgCl_2_, Na_2_SO_4_, to NaCl, respectively. Based on the Donnan exclusion mechanism, the rejection of a charged membrane increases with increasing co‐ion charges or decreasing counterion charges of salt solutions [41, 42]. In this case, the positively charged (PEI/SL)_7_‐GA membrane exhibits stronger repulsive interactions to Mg^2+^ ions than Na^+^ ions, and stronger attractive interactions to SO_4_^2–^ ions than Cl^–^ ions. So the Na^+^ or SO_4_^2–^ ions tend to move toward and pass through the membrane. Once some ions pass through the membrane, some counterions must pass through the membrane together to keep the electroneutrality of feed and permeate. Therefore, the (PEI/SL)_7_‐GA membrane rejects MgCl_2_ ions higher than Na_2_SO_4_. The salt rejection is also affected by the steric hindrance mechanism, as the Na^+^ and Cl^–^ ions with the smaller sizes may pass through the dense (PEI/SL)_7_‐GA membrane easier than the Mg^2+^ ions and SO_4_^2–^ ions. Thus, the membrane have the highest rejection for MgSO_4_ and the lowest rejection for NaCl. Therefore, the mechanism of ion rejection includs both of the Donnan exclusion and size exclusion mechanism. The movement of ions near a charged membrane surface is dependent on the interaction between ions and charges on the membrane surface, and multivalent ions possess stronger attractive or repulsive electrostatic interactions than monovalent ions.

To determin the molecular weight cutoff (MWCO) of the optimized (PEI/SL)_7_‐GA NF membrane, four cationic organic dyes with different molecular weight were used as solutes to investigate the dye rejections separately under the same operating conditions (the feed concentration of 0.1 g/L and the operating pressure of 1.0 MPa). As shown in Figure 6D, the dyes are neutral red (NR, 288.78 g/mol), Methylene Blue (MB, 319.85 g/mol), crystal violet (CV, 407.90 g/mol), Rhodamine B (RB, 479.01), and the rejection values are all higher than 90% except for NR. The MWCO value of the membrane is probably around 300 g/mol. The rejection results are presented in ascending order of 89.4% < 92.6 < 96.8% < 98.2%, which is in the same order of the molecular weight from low to high. It indicates that the separation mechanism for rejecting organic dye solutes with the molecular weight higher than 288 g/mol is primarily dominant by the steric hindrance mechanism. Therefore, the membrane may exhibit higher rejections for larger molecules.

The desalination performance of the optimized (PEI/SL)_7_‐GA membrane for divalent salts is compared with other polyelectrolytes LbL membranes reported in previous literature [13, 15, 33, 43, 44, 45, 46, 47, 48, 49]. Table 1 lists the membrane materials, the type of Mg^2+^ or Ca^2+^ salt, the salt concentration, the rejection, and the fluxes. Generally, the LbL membranes with more bilayer may have better rejection but lower fluxes, and most membranes listed in Table 1 have the rejection larger than 90% or the fluxes higher than 4.0 kg/(m^2^∙h∙bar). Although some literature reports higher rejections and higher fluxes than this work, it is worth to note that only two membranes were prepared from natural polyelectrolyte materials, SL in the (PEI/SL)_7_‐GA membrane and sodium carboxymethyl cellulose in the PEI/CMCNa membrane. Moreover, the (PEI/SL)_7_‐GA membrane exhibits the rejection of 95.3% and flux of 3.96 kg /(m^2^∙h∙bar) for separating MgSO_4_ solution with the concentration of 4.0 g/L, while the desalination performance of other membranes mostly for divalent salts solutions with the concentration not higher than 1.0 g/L. Therefore, the NF performance of the (PEI/SL)_7_‐GA is comparable with other LbL NF membranes prepared from traditional synthetic polyelectrolyte materials. It demonstrates that SL can be used as a natural polyelectrolyte as an alternative to the synthetic ones for fabricating novel LbL membranes with a promising prospect in water desalination. We anticipate the SL‐based LbL membrane can be further optimized in order to achieve high NF performance, for example, controlling the content of sulfonate groups in SL, chemically modifying SL, incorporating porous materials, using other polyelectrolytes or crosslinking agents, and so on.

**TABLE 1:** Desalination performance of polyelectrolyte LbL self‐assembly NF membranes for Mg^2+^ or Ca^2+^ salts

Membrane	Divalent salt	Salt concentration (g/L)	Rejection (%)	Permeance [L/(m^2^∙h∙bar)]	References
(PVA/PVS)_60_	MgSO_4_	1.0 mM	100	0.112	[13]
[PSS/PAH]_8_	MgSO_4_	5.0 mM	98	6.2	[43]
(PSS/PAH)_5_ on porous alumina	MgSO_4_	1.0	96	9.55	[44]
(PEI/SL)_7_‐GA	MgSO_4_	4.0	95.3	3.96	This work
[PSS/PAH]_4_	MgSO_4_	1.0	93.6	4	[15]
((PEI‐modified GO)/PAA)_5_/PVA‐GA	MgSO_4_	/	92.6	0.81	[45]
PEI/TMA	MgCl_2_	0.1	>90	23.5	[46]
(PEI/SCF)_4.5_	CaCl_2_	0.5	90.6	4.49	[47]
(PEI/PAA)/PVA‐GA	MgSO_4_	/	83.	0.78	[45]
[PAH/PSS]_1_PAH/PSSMA	MgSO_4_	1.0	86.4	7.24	[48]
(PDDA/GO)_4_	MgSO_4_	/	69.2	6.95	[49]
PEI/CMCNa	MgSO_4_	0.5	19.2	14.00	[33]

## 4 CONCLUDING REMARKS

Novel (PEI/SL)_7_‐GA NF membranes were prepared by PEI and SL via LbL self‐assembly and crosslinked by GA. The characterization results indicate that the (PEI/SL)_7_ bilayers can be successfully deposited on the support membranes and the (PEI/SL)_7_‐GA membranes having dense and hydrophilic selective layers. The NF performance for removing MgSO_4_ from water shows that the increase in GA concentration and crosslinking time can increase the membrane selectivity but decrease the membrane permeance. Adding NaCl supporting electrolytes to fabricate the (PEI/SL)_7_‐GA membranes tend to decrease the membrane selectivity but increases the membrane permeance. The optimized (PEI/SL)_7_‐GA membrane exhibit higher rejections for Mg^2+^ salts than Na^+^ salts, and has the MWCO properties around 300 g/mol evaluated by organic dye solutes. The (PEI/SL)_7_‐GA membrane shows a promising potential for water desalination, especially for separating high concentration salt solution. It provides a new way to valorize the underutilized, renewable, and low‐cost SL waste.

## CONFLICT OF INTEREST

The authors have declared no conflict of interest.

## Supporting information

Supporting Information

## Data Availability

The data used to support the findings of this study are available from the corresponding author upon request.
