# Supplementary material for: Preparation of lignosulfonate‐based nanofiltration membranes with improved water desalination performance
Source: Eng Life Sci. 2021 Apr 2;21(6):417–28. doi: 10.1002/elsc.202000102 (PMC8182288; doi:10.1002/elsc.202000102)
Supplement: Supplementary file 1 — Supporting Information [file ELSC-21-417-s001.pdf]

Supporting Information

Preparation of lignosulfonate-based nanofiltration membranes with improved water desalination performance

Wangqu Liu[1,2], Xin Geng[1], Saisai Li[1], Xia Zhan[3], Jiding Li[4], Luying Wang[*1] and Jiandu Lei[*1]

[1]Beijing Key Laboratory of Lignocellulosic Chemistry, Beijing Forestry University, Beijing 100083, China.

[2]Department of Chemical and Biomolecular Engineering, Johns Hopkins University, 3400 North Charles Street, Baltimore, Maryland 21218, United States

[3]Key Laboratory of Cleaner Production and Integrated Resource Utilization of China National Light Industry, Beijing Technology and Business University, Beijing 100048, China

[4]State Key Laboratory of Chemical Engineering, Department of Chemical Engineering, Tsinghua University, Beijing 100084, China

Correspondence: Dr. Luying Wang (wangly@bjfu.edu.cn), Dr. Jiandu Lei (ljd2012@bjfu.edu.cn). Beijing Key Laboratory of Lignocellulosic Chemistry, Beijing Forestry University, Beijing 100083, China.

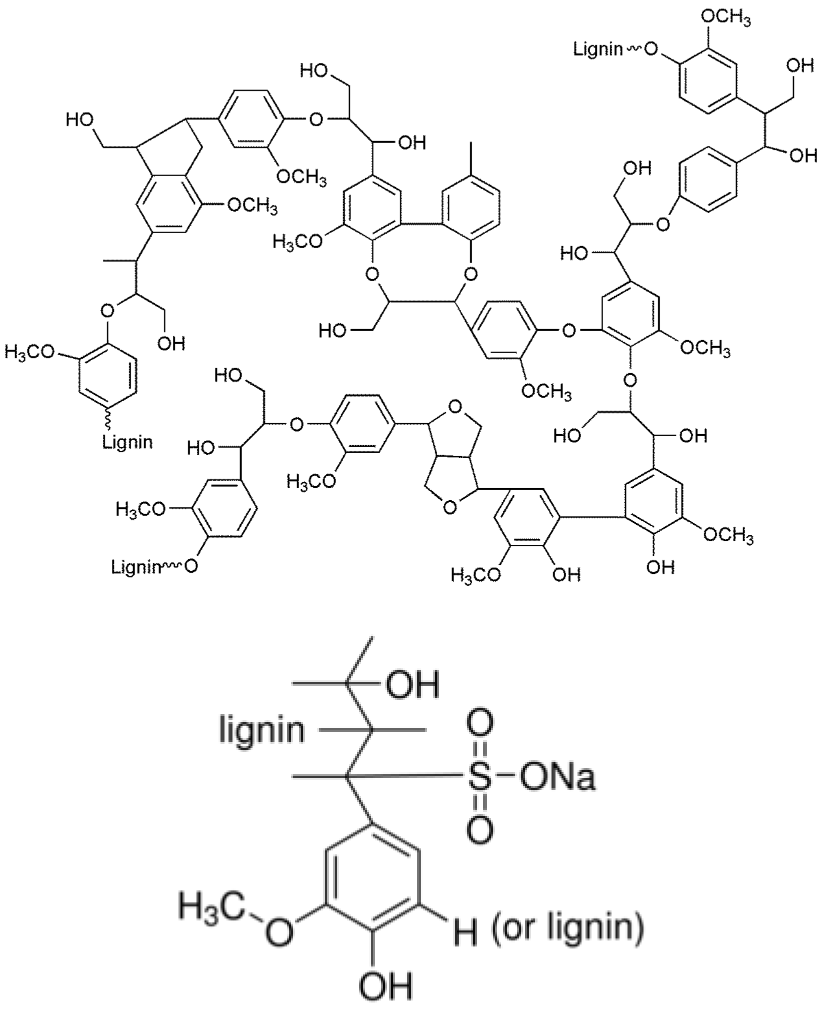

Figure S1. Molecular formulas of lignin and lignosulfonates.

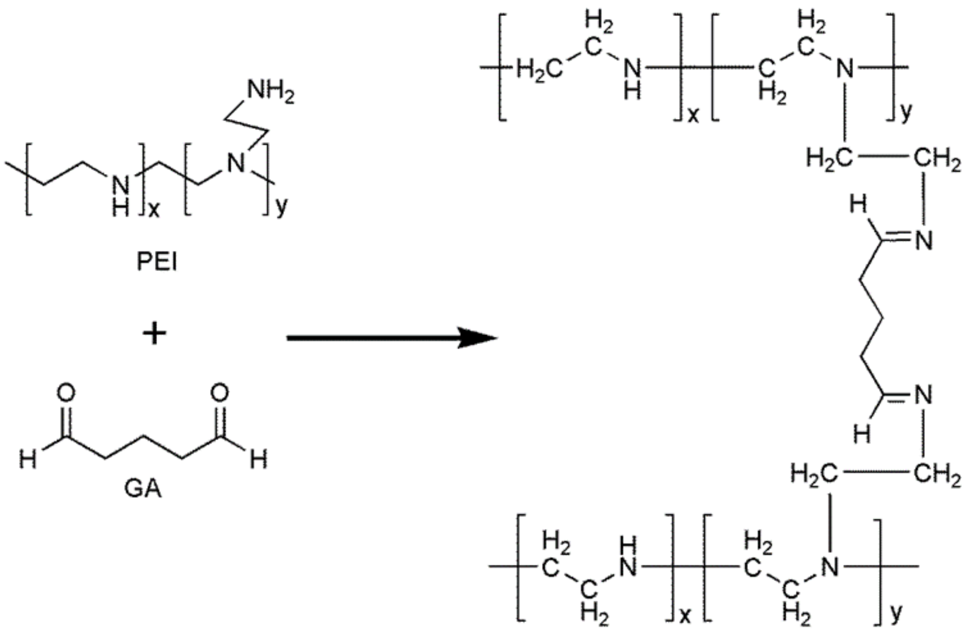

Figure S2. Crosslinking reaction between PEI and GA.

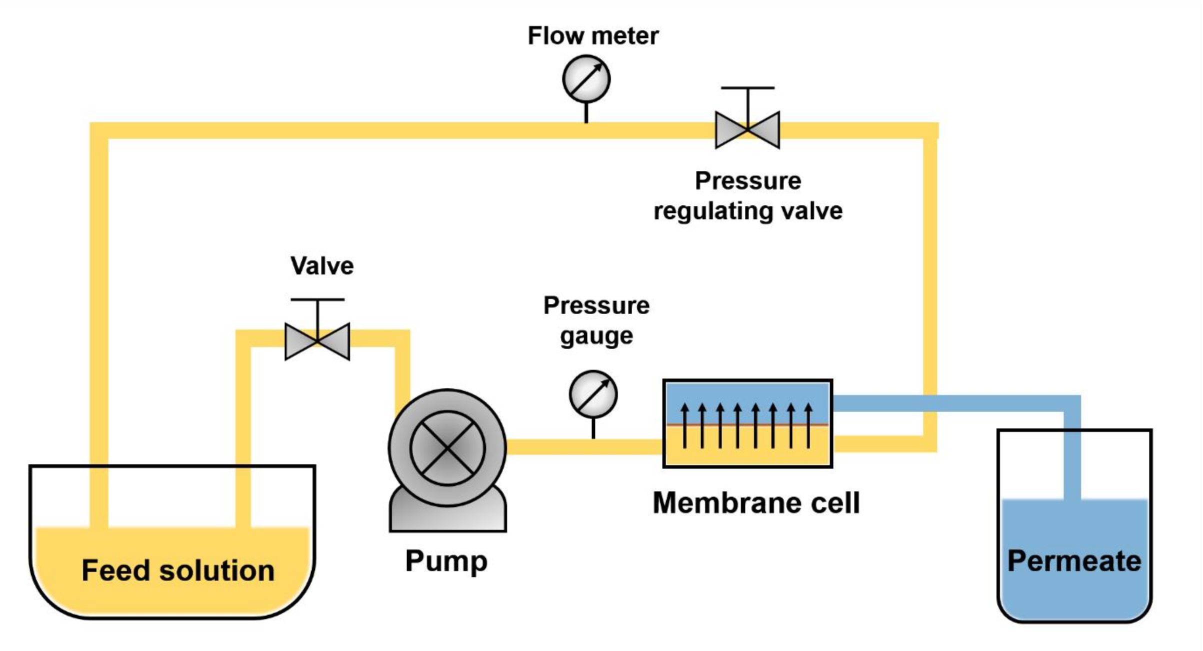

Figure S3. Schematic of the NF performance testing system.

Table S1. Water contact angle of $(PEI/SL)_7$-GA with different supporting electrolyte (NaCl) concentration

NaCl Concentration (M)	Contact angles (°)
0	64.6±1.9
0.25	57.5±2.8
0.50	55.5±3.1
0.75	56.0±1.8
1.0	57.6±3.3
